# Patient and Public Involvement (PPI) and Responsible Research and Innovation (RRI) approaches in mental health projects involving young people: a scoping review protocol

**DOI:** 10.1186/s40900-024-00591-1

**Published:** 2024-06-11

**Authors:** Josimar Antônio de Alcântara Mendes, Mathijs Lucassen, Alex Adams, Lucy Martin, Christine Aicardi, Rebecca Woodcock, Emma Nielsen, Ellen Townsend, Marina Jirotka

**Affiliations:** 1 Department of Computer Science, University of Oxford, 39a St. Giles, Oxford, OX1 3LW UK; 2 City, University of London, London, UK; 3 University of Nottingham, Sprouting Minds (Young People Advisory Group – Digital Youth), Nottingham, UK; 4 King’s College London, London, UK; 5 MindTech Patient and Public Involvement (PPI) Coordinator, University of Nottingham, Nottingham, UK; 6 Faculty of Medicine & Health Sciences, University of Nottingham, Nottingham, UK; 7 Faculty of Science, University of Nottingham, Nottingham, UK; 8 University of Oxford, Oxford, UK

**Keywords:** Mental health, Patient and public involvement, Responsible research and innovation, Young people, Youth participation

## Abstract

**Background:**

Rather than being perceived as merely ‘part of the problem’, the perspectives and experiences of young people play a pivotal role in devising effective solutions for mental health challenges. Two distinct methodologies that aid in this endeavour are ‘patient and public involvement’ (PPI) and ‘responsible research and innovation’ (RRI). However, there is a tendency to conflate PPI and RRI practices, leading to ambiguity in their application. Moreover, the extent and nature of young people’s involvement in mental health-related projects (namely: research, intervention, product development) employing these methodologies, and the subsequent implications thereof, remain unclear. Consequently, the proposed scoping review aims to identify and analyse literature pertaining to PPI and RRI approaches in mental health projects that engage young people in collaboration.

**Methods:**

The selected databases will be MEDLINE, PsycINFO, PsycArticles, Scopus, Web of Science, IBBS, CINAHL (EBSCO) and ASSIA. Comprehensive searches will span from the inception of each database. A pilot test will be conducted to assess the screening criteria and data extraction form, with two authors independently reviewing titles and abstracts. Full-text articles meeting the inclusion criteria will undergo narrative syntheses, with results presented in tabular format. Feedback on the findings from a youth perspective will be sought from young people within our broader research network, namely Sprouting Minds. The review will adhere to the guidelines outlined by the Joanna Briggs Institute (JBI) and follow the PRISMA-ScR procedures. Inclusion criteria will comprise English-language, primary research peer-reviewed articles focused on Patient and Public Involvement (PPI) or Responsible Research and Innovation (RRI), examining mental health-related research processes, interventions, and products developed in collaboration with young people. Studies employing quantitative, qualitative, and mixed-methods approaches will be considered, while non-journal publications will be excluded.

**Discussion:**

The intended scoping review aims to map the literature concerning mental health-related projects that engage with young people through PPI or RRI approaches. The outcomes hold promise for enriching the participatory research domain, particularly in studies centred on young people and their mental well-being. Furthermore, by delineating potential overlaps and distinctions between PPI and RRI, the findings stand to aid mental health researchers and practitioners in making informed decisions about the most suitable approach for their projects when partnering with young individuals.

**Systematic review registration:**

Open Science Framework (registration: DOI 10.17605/OSF.IO/N4EDB).

**Supplementary Information:**

The online version contains supplementary material available at 10.1186/s40900-024-00591-1.

## Abstract (plain language summary)

This study aims to understand how young people are involved in mental health projects (namely: research, intervention, and product development) and how their perspectives shape the outcomes. Two approaches, Patient and Public Involvement (PPI) and Responsible Research and Innovation (RRI), are often used in these projects, but it’s not clear how they differ or how young people are involved. To explore this, the researchers will review existing literature on mental health projects involving young people. They will search databases for articles that describe or evaluate these projects, focusing on how young people are involved in the research and/or development processes. They will include studies in English that have been peer-reviewed and cover both numerical and text-based research. Young people will be involved to provide feedback from their perspective. This review will follow established guidelines to ensure trustworthiness and transparency. The findings from this review will help researchers and professionals understand the best ways to involve young people in mental health projects. By clarifying the differences between PPI and RRI and highlighting effective strategies, this study aims to improve future research and ultimately benefit young people’s mental health.

**Supplementary Information:**

The online version contains supplementary material available at 10.1186/s40900-024-00591-1.

## Background

The existing literature on collaborative projects (namely: research, intervention, product development) involving young people and mental health is notably limited, particularly concerning the depth and breadth of youth engagement [1]. Within this context, both ‘Patient and Public Involvement’ (PPI) and ‘Responsible Research and Innovation’ (RRI) approaches have emerged to structure and inform various health-related initiatives seeking insights from young people’s lived experiences and expertise [2–5].

The UK National Institute for Health and Care Research (NIHR) outlines PPI as “an active partnership between patients and the public and researchers in the research process, rather than the use of people as ‘subjects’ of research” and that PPI “is often defined as doing research ‘with’ or ‘by’ people who use services rather than ‘to’, ‘about’ or ‘for’ them”^1^. There are various definitions and implications for PPI, generally centred around the concept of active and transformative participation by the public and patients. However, PPI definitions and practices can often be ‘messy‘ [6].

Similar to the case of PPI, the definition of RRI is also diverse and plural, lacking consensus [7, 8]. For this study, we will consider RRI as research and innovation endeavours that responsibly foster creativity in science and technology by anticipating and mitigating unintended impacts and/or risks, considering what is socially desirable and in the public interest, while considering the views and needs of key stakeholders [9].

The distinction between PPI and RRI can be blurred too, as they are often perceived as similar practices with different names, especially across different fields and funding bodies, and they may manifest differently even under the same terminology, particularly in the case of PPI [10]. Consequently, merely adopting participatory terminology may not suffice to identify and endorse effective practices, a concern heightened within the realm of mental health research [11]. Traditionally, the domain of mental health research has not embraced RRI terminology, possibly due to its absence from research culture or the primary requirements of funders. Nonetheless, certain practices, such as co-design and co-production, may embody RRI principles in different contexts, underscoring the complexity of terminology within this field.

### A glance at the review’s purpose

PPI and RRI are inherently context-dependent approaches [6, 12] yet they are often employed interchangeably within collaborative projects. This prompts critical inquiries: how do mental health-related projects (namely: research, intervention, product development) navigate the realms of PPI and RRI when engaging with young people? What are the potential ramifications of adopting PPI or RRI within these contexts? Are there established best practices for implementing these approaches in such settings? These questions underscore the necessity of gaining a deeper comprehension of collaborative processes involving young people in the mental health domain [1], thus providing a foundational rationale for the proposed scoping review.

Another important issue is the need to reflect on the ‘participatory approach’ itself [10, 11, 13]. The 12th article of the United Nations Convention on the Rights of the Child states that children and young people have the right to freely express their views, wishes and feelings in all matters affecting them [14]. PPI and RRI serve as avenues for upholding this fundamental right. Furthermore, these approaches have the potential to bolster the development of more ecologically valid and robust research and innovation initiatives. However, in the absence of proper recognition of PPI or RRI within such contexts, the efficacy of these approaches may be compromised or even undermined. Therefore, it is imperative to delineate how mental health-related projects are addressing youth participation in accordance with PPI and RRI principles, not only to safeguard children’s rights but also to enhance the application of these approaches in the mental health arena and associated innovation endeavours.

## Methods/design

This scoping review aims to identify and map literature concerning PPI and RRI approaches in mental health-related projects (namely: research, intervention, product development) involving collaborations with young people. The research questions are: (I) how does the literature define the PPI and RRI approaches in mental-health-related projects involving young people? (II) what is the type and extent of young people’s involvement in PPI and RRI mental-health-related projects?

The proposed scoping review will be conducted following the Joanna Briggs Institute (JBI) methodology for scoping reviews [15] as well as the PRISMA-ScR protocol [16]. This review protocol has been registered with Open Science Framework (DOI 10.17605/OSF.IO/N4EDB) on the 13th of December 2023.

### Inclusion and exclusion criteria

Following the JBI guidance [15], the inclusion and exclusion criteria are divided in terms of population, concept, context and sources – see Table 1.

**Table 1:** PCCS acronym and inclusion and exclusion criteria

	CRITERIA	
CATEGORY	Inclusion	Exclusion
*Population*	Articles describing and/or evaluating mental health-related research processes and product or intervention development in collaboration with young people. We will consider ‘young people’ in a broad sense – ranging from the early stages of adolescence (namely, 10 years old) to early adulthood (namely, until 24 years old) [17].	Articles focusing on other population rather than on young people (as we operationalised)
*Concept*	Articles that explore and discuss the concepts and the processes of the ‘patient and public involvement’ (PPI) and/or ‘responsible research and innovation’ (RRI) approaches. These include, but are not limited to, face-to-face, online and hybrid mental health-related research processes and product or intervention development in collaboration with young people	Articles not focusing on PPI or RRI by just mentioning them *en passant*
*Context*	Articles reporting mental health-related research processes and product or intervention development in collaboration with young people	Articles not focusing on mental health-related research or product development processes
*Sources* *(Type of studies)*	This scoping review will include only primary studies articles peer-reviewed and published in English with no time restriction – namely, we will retrieve databases’ articles since their inception. We will include quantitative, qualitative, and mixed methods	The review will exclude prospective study protocols, review articles, and general grey work

## Search strategy

This review will employ a two-fold search strategy. Firstly, an initial limited search of PsycARTICLES, PsycINFO, and MEDLINE databases was conducted to identify relevant articles on the topic – please, see Appendix I to check descriptors and strings and Appendix II for the results of this initial search, which was supported by a subject expert librarian. The text words in the title and abstract of the retrieved papers, as well as the index terms used to describe the articles, will inform the second step. This will involve a comprehensive search across multiple databases, including MEDLINE, PsycINFO, PsycArticles, Scopus, Web of Science, IBBS, CINAHL (EBSCO), and ASSIA. The search strategy, incorporating all identified keywords and index terms, will be tailored for each information source.

## Study/source of evidence selection

**Fig. 1:**
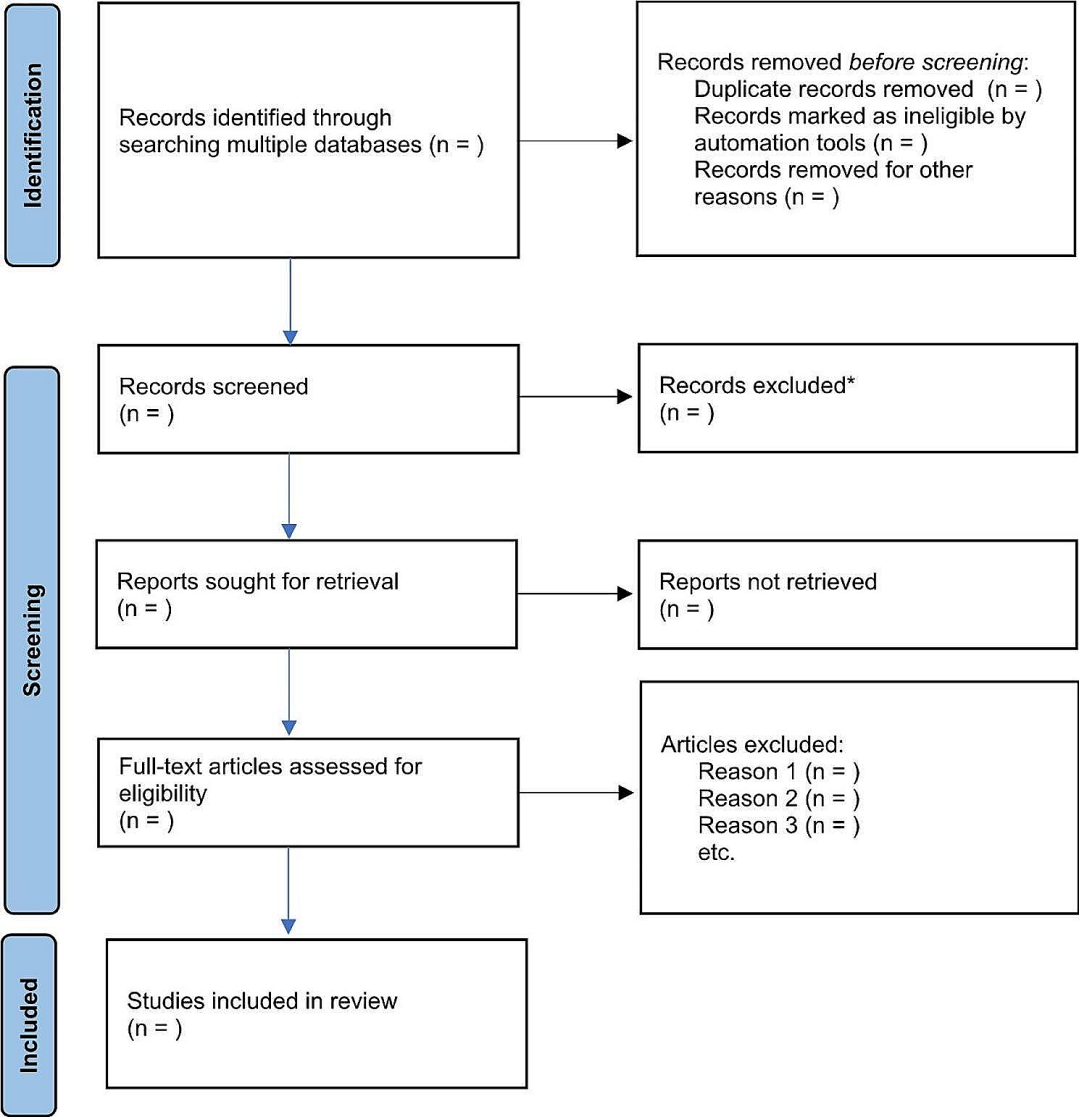
Selection of Sources of Evidence Process According to PRISMA and adapted from [16] and [20]

Following the search, all identified records will be collated and uploaded into CADIMA – an open platform aimed at guiding the conduction and furnishing the documentation of systematic reviews and scoping reviews [18]. CADIMA assists in managing the entire review process. For instance, it enhances the interrater consistency check, facilitates the study selection phase, and supports the appraisal stage. Assisted by CADIMA, we will remove duplicated results. The first selection phase will be focused on the studies’ titles and abstracts. Before screening titles and abstracts, we will run a consistency check with a randomised sample of 5% of the results to appraise the reviewers’ levels of agreement and inter-reliability. This process will be aided by CADIMA, which will also automatically run a Kappa test for inter-rater reliability [19] – reviewers will only start screening when a statistically significant level of agreement of 75% (or greater) is achieved [15]. After that, 100% of the results will be independently screened by two reviewers in parallel. In the next phase, a randomised sample of 10% of selected studies’ full-text will be assessed in detail against the inclusion criteria by 2 independent reviewers – CADIMA will also automatically run a Kappa test for this inter-rating task. Then, 100% of selected studies will be independently assessed by two reviewers in parallel. Any disagreements that arise between the reviewers at each stage of the selection process will be resolved through discussion or with a third reviewer. Reasons for exclusion of full-text papers that do not meet the inclusion criteria will be recorded and reported in the scoping review. The results of the search will be reported in full in the final scoping review and presented in a PRISMA flow diagram – see Fig. 1.

### Data extraction

Data will be extracted from articles included in the scoping review using a data extraction tool developed by the review team. The data extracted will include specific details concerning three domains: (1) *general descriptions and methods* (for instance, year of publication; research design); (2) *involvement characterisation* (for instance, studies’ specific mental health topic); and (3) *operational definitions for involvement* (for instance, articles’ PPI and RRI definitions and its implications) – a preliminary data extraction tool is presented in Table 2. The draft data extraction tool will be modified and revised as necessary during the process of extracting data from each included paper. Modifications will be detailed in the full scoping review. Any disagreements that arise between the reviewers will be resolved through discussion or with a third reviewer. Authors of papers will be contacted to request missing or additional data, where required.

**Table 2:** Preliminary Data Extraction Tool

Category	Data Extracted
*1. General descriptions and methods*	1.1. Authors: _________ YP as co-authors: Yes No 1.2. Year: _________ 1.3. Place: _________ 1.4. Collaborative Approach: PPI RRI 1.5. Range age of young people involved: _________ 1.6. Mean age of young people involved: _________ Type of design: 1.7. Qualitative (descriptive, e.g., ethnography) 1.8. Qualitative interviews with experts (e.g., professionals and practitioners) 1.9. Qualitative interviews with young people Both 1.6. and 1.7. 1.10. Quantitative. Specify: _________ 1.11. Mixed Methods
*2. Involvement characterisation*	2.1. Study’s specific mental health topic: Type and extensiveness of young people’s involvement: 2.1. Co-design 2.2. Co-production 2.3. Consultive 2.4. Other: _________
*3. Operational definitions for involvement*	3.1. Study’s PPI definition, if any: 3.2. Study’s RRI definition, if any:

### Data analysis and presentation

The data will be presented in tabular or graphical format, where appropriate. Definitions of PPI and RRI, as well as definitions concerning the type of young people’s involvement, will be mapped in more detail – for instance, through a narrative summary. Once the results are charted, a group of young people will critique them from their perspective. The outcomes of this review will be published in a peer-reviewed journal.

## Discussion

A preliminary MEDLINE (Ovid) search of ‘PPI’ or ‘RRI’ or ‘co-design’ or ‘co-creation’ yielded 4,006 (namely, concept 1) in October 2023. In relation to ‘mental health’ as a term, there were 276,525 (namely, concept 2) results. When these two (namely, concepts 1 and 2) were combined, 363 papers were identified. These included a range of highly relevant papers, such as ‘Exploring the feasibility of a mental health application (JoyPopTM) for Indigenous youth’.

A preliminary search of PROSPERO, MEDLINE, the Cochrane Database of Systematic Reviews, and JBI Evidence Synthesis was conducted, and no current or in-progress scoping reviews or systematic reviews on the particular topic we have outlined were identified. However, several related reviews were found in PROSPERO. For instance, “A meta-synthesis of methods and approaches to co-production when designing, producing and evaluating smartphone applications for mental healthcare [CRD42023414007]” and “A systematic review of the models of co-production used in mental health research [CRD42020208780]” were among them. Neither of these reviews has an explicit focus on young people. Furthermore, “A systematic review of approaches to patient and public involvement in mental health research with young people (11–20 years) [CRD42020171476]” explores the literature in relation to young people, but its emphasis is on PPI alone (namely, it does not include RRI), and its final report has not yet been published despite being registered in 2020.

The findings from these searches underscore the imperative of conducting the proposed scoping review. Moreover, we acknowledge the vital principle of “*nothing about us without us*”. As such, our review team includes young people from our wider research network, who will serve as co-authors and contribute to critiquing the results from a young person’s perspective. This approach represents a significant departure from traditional systematic reviews in the field, emphasising inclusivity and diverse viewpoints. Their involvement is pivotal not only to uphold their right to participate but also to offer an ecological perspective throughout the review process. Ultimately, our objective is to achieve a comprehensive and insightful mapping of how PPI and RRI are addressed in mental health-related projects involving young people.

We believe that the outcomes of the proposed review will yield valuable insights for the participatory research domain, particularly in studies focusing on young people and their mental health. Additionally, by delineating potential overlaps and distinctions between PPI and RRI, our findings could assist mental health researchers and professionals in making well-informed decisions about the most suitable approach for their projects when engaging with young people.

### Potential impact of the review at micro, meso, and macro levels

At the micro level, this scoping review will have the potential to enhance individual and interpersonal outcomes in collaborative projects (namely: research, intervention, and product development) involving young people and in the mental health field. Hence, the review will potentially identify best practices for engaging young people in mental health-related projects, which can lead to: (1) *empowerment of young people*: by delineating effective PPI and RRI approaches, the review can help to empower the youth by ensuring their voices are heard and valued in mental health-related projects; (2) *enhancing communication and collaborative processes*: the review can provide insights into how to facilitate better communication and collaboration between researchers/developers and young people involved. Improved interactions can lead to richer data collection and a deeper understanding of young people’s mental health needs [1]; (3) *tailored interventions*: understanding the nuances of youth participation can inform the development of more personalised and more effective mental health interventions/products. This can lead to better outcomes for young people who directly benefit from these projects.

At the meso level, the findings of this scoping review can instrumentalise a more responsible and inclusive involvement of young people in organisations and communities in the field of mental health. In this sense, the outcomes of this review can aid organisations in the process of adopting best practices for PPI and RRI, leading to more inclusive and effective collaborative research methodologies. We understand these outcomes could also help to better shape processes of community engagement as they might help to make a case for the importance of involving young people in mental health projects, thereby fostering a culture of inclusivity and shared decision-making within communities.

At the macro level, this scoping review has the potential to influence broader societal and policy frameworks. By providing a comprehensive overview of how PPI and RRI are implemented in mental health projects involving young people, the review can inform policymakers about the importance of these approaches. This can lead to the development of policies that mandate youth participation in mental health research and innovation, for instance. The emphasis on participatory approaches can foster a cultural shift towards recognizing and valuing the contributions of young people in mental health projects. This can challenge traditional power dynamics and promote a more democratic and participatory research culture.

### Electronic supplementary material

Below is the link to the electronic supplementary material.

Supplementary Material 1

Supplementary Material 2

Supplementary Material 3

## Data Availability

No datasets were generated or analysed during the current study.

## Footnotes

1 Source https://www.nihr.ac.uk/about-us/glossary.htm?letter=P#:~:text=An%20active%20partnership%20between%20patients,'%20or%20’for’%20them.

## Abbreviations

CADIMA: Central Access Database for Impact Assessment of crop genetic improvement technologies
PCCS: Participants, Concept, Context and Sources
PPI: Patient and Public Involvement
PRISMA: Preferred Reporting Items for Systematic Reviews and Meta-Analyses
PRISMA-ScR: PRISMA extension for scoping reviews
PROSPERO: The International Prospective Register of Systematic Reviews
RRI: Responsible Research and Innovation
