# Supplementary material for: Patient and Public Involvement (PPI) and Responsible Research and Innovation (RRI) approaches in mental health projects involving young people: a scoping review protocol
Source: Res Involv Engagem. 2024 Jun 11;10:60. doi: 10.1186/s40900-024-00591-1 (PMC11167781; doi:10.1186/s40900-024-00591-1)
Supplement: Supplementary file 1 — Supplementary Material 1 [file 40900_2024_591_MOESM1_ESM.docx]

# Appendix I: Search Strategy

|  | **POPULATION** | **CONCEPT/CONTENT** | **CONTEXT** |
| --- | --- | --- | --- |
| **PPI** | “young people” OR adolescen* OR youth OR teenage* OR teens | “patient and public involvement” OR “patient involve*” OR “patient engage*” OR “user involve*” OR “user engage*” OR “participant engage*” OR “participant involve*” OR  “public engage*” OR “public involve*” OR “expert by experience” OR “co-design*” OR codesign* OR “co-produc*” OR coproduc* OR co-creat* OR cocreat* | “mental health” OR  “mental wel*” OR “emotional health” OR “emotional wel*” OR “psychological health” OR “psychological wel*”  OR “mental health service”  OR “mental health care” OR “psycholog*” OR “psychiat*” |
| **RRI** | “young people” OR adolescen* OR youth OR teenage* OR teens | “responsible research and innovation” OR “responsible innovat*” OR “responsible engagement” OR “responsible involvement” OR “responsible research” OR “participat* research” OR “peer research” OR “citizen science” OR “citizen participation” OR human-cent* OR “participat* design” OR “technology assess*” OR “ethical, legal and social aspects” OR “ethical, legal, and social implications” OR “user-led” OR “user-cent*” OR “client-led” or “patient-led” OR “client-cent*” OR “patient-cent*” | “mental health” OR  “mental wel*” “emotional health” OR “emotional wel*” OR “psychological health” OR “psychological wel*”  OR “mental health service”  OR “mental health care” OR “psycholog*” OR “psychiat*” |
