# Supplementary material for: Patient and Public Involvement (PPI) and Responsible Research and Innovation (RRI) approaches in mental health projects involving young people: a scoping review protocol
Source: Res Involv Engagem. 2024 Jun 11;10:60. doi: 10.1186/s40900-024-00591-1 (PMC11167781; doi:10.1186/s40900-024-00591-1)
Supplement: Supplementary file 2 — Supplementary Material 2 [file 40900_2024_591_MOESM2_ESM.docx]

# Appendix II: Initial Limited Search

## Database: PsychARTICLES

Search conducted on December, 2023.

| **Search** | **Query** | **Records retrieved** |
| --- | --- | --- |
| #1 | “young people” OR “adolescent” OR “youth” OR “teenagers” OR “teens” | 58,049 |
| #2 | “patient and public involvement” OR “patient involvement” OR “patient engagement” OR “user involvement” OR “user engagement” OR “participant engagement” OR “participant involvement” OR “public engagement” OR “public involvement” OR “expert by experience” OR “co-design” OR “codesign” OR “co-produc*” OR “coproduc*” OR “co-creat*” OR “cocreat*” | 2,278 |
| #3 | “responsible research and innovation” OR “responsible innovation” OR “responsible engagement” OR “responsible involvement” OR “responsible research” OR “participatory research” OR “peer research” OR “citizen science” OR “citizen participation” OR “human-centered” OR “human-centered” OR “participatory design” OR “technology assessment” OR “ethical, legal and social aspects” OR “ethical, legal, and social implications” OR “user-led” OR “user-centred” OR “user-centered” OR “client-led” or “patient-led” OR “client-centred” OR “client-centered” OR “patient-centered” OR “patient-centred” | 59,61 |
| #4 | “mental health” OR “mental well-being” OR “mental welfare” OR “emotional health” OR “emotional well-being” OR “psychological health” OR “psychological well-being” OR “mental wellness” OR “emotional wellness” OR “mental health service” OR “mental health care” OR “psychology” OR “psychological” OR “psychiatry” | 235,062 |
| #5 | #1 AND #2 AND #4 | 991 |
| #6 | #1 AND #3 AND #4 | 2,581 |
| Limited to December 2023; English | |  |

## Database: PsychINFO

Search conducted on December, 2023.

| **Search** | **Query** | **Records retrieved** |
| --- | --- | --- |
| #1 | “young people” OR “adolescent” OR “youth” OR “teenagers” OR “teens” | 544,918 |
| #2 | “patient and public involvement” OR “patient involvement” OR “patient engagement” OR “user involvement” OR “user engagement” OR “participant engagement” OR “participant involvement” OR “public engagement” OR “public involvement” OR “expert by experience” OR “co-design” OR “codesign” OR “co-produc*” OR “coproduc*” OR “co-creat*” OR “cocreat*” | 16,621 |
| #3 | “responsible research and innovation” OR “responsible innovation” OR “responsible engagement” OR “responsible involvement” OR “responsible research” OR “participatory research” OR “peer research” OR “citizen science” OR “citizen participation” OR “human-centered” OR “human-centered” OR “participatory design” OR “technology assessment” OR “ethical, legal and social aspects” OR “ethical, legal, and social implications” OR “user-led” OR “user-centred” OR “user-centered” OR “client-led” or “patient-led” OR “client-centred” OR “client-centered” OR “patient-centered” OR “patient-centred” | 31,680 |
| #4 | “mental health” OR “mental well-being” OR “mental welfare” OR “emotional health” OR “emotional well-being” OR “psychological health” OR “psychological well-being” OR “mental wellness” OR “emotional wellness” OR “mental health service” OR “mental health care” OR “psychology” OR “psychological” OR “psychiatry” | 3,355,379 |
| #5 | #1 AND #2 AND #4 | 782 |
| #6 | #1 AND #3 AND #4 | 2,350 |
| Limited to December 2023; English | |  |

## Database: MEDLINE

Search conducted on December, 2023.

| **Search** | **Query** | **Records retrieved** |
| --- | --- | --- |
| #1 | “young people” OR “adolescent” OR “youth” OR “teenagers” OR “teens” | 1,972,493 |
| #2 | “patient and public involvement” OR “patient involvement” OR “patient engagement” OR “user involvement” OR “user engagement” OR “participant engagement” OR “participant involvement” OR “public engagement” OR “public involvement” OR “expert by experience” OR “co-design” OR “codesign” OR “co-produc*” OR “coproduc*” OR “co-creat*” OR “cocreat*” | 29,306 |
| #3 | “responsible research and innovation” OR “responsible innovation” OR “responsible engagement” OR “responsible involvement” OR “responsible research” OR “participatory research” OR “peer research” OR “citizen science” OR “citizen participation” OR “human-centered” OR “human-centered” OR “participatory design” OR “technology assessment” OR “ethical, legal and social aspects” OR “ethical, legal, and social implications” OR “user-led” OR “user-centred” OR “user-centered” OR “client-led” or “patient-led” OR “client-centred” OR “client-centered” OR “patient-centered” OR “patient-centred” | 91,618 |
| #4 | “mental health” OR “mental well-being” OR “mental welfare” OR “emotional health” OR “emotional well-being” OR “psychological health” OR “psychological well-being” OR “mental wellness” OR “emotional wellness” OR “mental health service” OR “mental health care” OR “psychology” OR “psychological” OR “psychiatry” | 2,570,329 |
| #5 | #1 AND #2 AND #4 | 1,324 |
| #6 | #1 AND #3 AND #4 | 3,510 |
| Limited to December 2023; English | |  |
